# A receptor dependent-*4D QSAR* approach to predict the activity of mutated enzymes

**DOI:** 10.1038/s41598-017-06625-x

**Published:** 2017-07-24

**Authors:** R. Pravin Kumar, Naveen Kulkarni

**Affiliations:** Polyclone Bioservices, #437, 40th Cross, Jayanagar 5th Block, Bangalore, 560041 India

## Abstract

Screening and selection tools to obtain focused libraries play a key role in successfully engineering enzymes of desired qualities. The quality of screening depends on efficient assays; however, a focused library generated with a priori information plays a major role in effectively identifying the right enzyme. As a proof of concept, for the first time, receptor dependent – 4D Quantitative Structure Activity Relationship (RD-4D-QSAR) has been implemented to predict kinetic properties of an enzyme. The novelty of this study is that the mutated enzymes also form a part of the training data set. The mutations were modeled in a serine protease and molecular dynamics simulations were conducted to derive enzyme-substrate (E-S) conformations. The E-S conformations were enclosed in a high resolution grid consisting of 156,250 grid points that stores interaction energies to generate QSAR models to predict the enzyme activity. The QSAR predictions showed similar results as reported in the kinetic studies with >80% specificity and >50% sensitivity revealing that the top ranked models unambiguously differentiated enzymes with high and low activity. The interaction energy descriptors of the best QSAR model were used to identify residues responsible for enzymatic activity and substrate specificity.

## Introduction

Screening and selection process is the crucial step in making focused libraries to derive novel properties of industrial enzymes. Rational redesign^1–3^ and directed evolution^4, 5^ or a combination of the two has proven to be successful in obtaining enzyme of desired properties in pharma, biotech, brewery, textile, chemical, dairy, tannery, food processing and other process intensive industries^6, 7^. However, these methods have their own limitations. Rational redesign relies on the sequence homology for amino acid replacement which in many cases does not consider the structural properties of the protein and directed evolution is constrained by low-throughput and requires an efficient assay for screening large number of potential mutants^8^. Present day engineering protocols are designed to have low-throughput screening techniques that capture highly specific features of an enzyme^9^. The success of obtaining smaller, highly qualified libraries depends on the functional diversity based on the protein sequences and efficient screening & selection assays used to filter mutants. To this end, different approaches such as *insilico* thermodynamic & steric structural considerations of the enzyme-substrate complex, *in vitro* mutagenesis experiments and even activity profiles from initial rounds of directed evolution experiments have been used to attain considerable success rate^10–13^. Recently, multivariate statistical techniques have been applied to model protein sequence–function relationships and guide the evolutionary process by rapidly identifying beneficial diversity for recombination^14^.

Powerful computational methods such as molecular dynamics (MD) and quantum mechanics/molecular mechanics (QM/MM) approaches are used to study and engineer enzymes^15^. MD simulations of TS analogs for screening enables enzyme engineering^16^ and short MD simulations of near attack configuration helps to evaluate enzyme enantioselectivity^17^. As an alternative, a rapid and robust approach to predict enzyme activity with large number of substrates using mechanism-based geometry criteria in combination with molecular docking was developed^18^. Short simulations are integrated in quantitative structure activity relationship (QSAR) protocol to predict biological activities such as pIC50 of small molecules (inhibitors)^19^. QSAR studies were also implemented to predict kinetic properties of enzymes^20^. Comparative binding energy (COMBINE) analysis was conducted for 18 substrates of the haloalkane dehalogenase to identify the amino acid residues determining the substrate specificity of the haloalkane dehalogenase. Also, QSAR models built on subjects where enzyme interacts with different substrates were used to predict activity of enzyme variants^21^. Two parameters, highest occupied molecular orbital derived using QM simulations and atomic distance between reactive groups were used as descriptors to build QSAR model to predict *K*_*cat*_ values of horseradish peroxidase^22^. Later this distance parameter was used to filter mutations with better activity^23^. In all incidences where QSAR was used to predict the kinetic properties of enzymes and filter mutations the variants of the enzyme were not included in the training data set of the QSAR study. The mutations were incorporated in the structure of the enzyme only for external validations. Perhaps the most intriguing question is that, how reliable are the predictions of a QSAR study that does not include enzyme variants for generating the model? Presumably, the QSAR protocol should capture variables of an E-S reaction as and when the mutations are incorporated in the enzyme and all other components in the E-S reaction remains the same. Capturing the details of per atomic changes (motion & energy) in an E-S reaction as a result of mutations, derived from simulation studies can improve the accuracy of activity predictions. Apparently, this is more appropriate and rational than just using the information on the changes in the substrate for screening enzyme mutations. This is because the constants of the QSAR models for each spatial QSAR descriptor are derived from the changing E-S dynamics as a result of mutation in the enzyme. Herein, for the first time we have demonstrated an effective method that; a) implements the RD-4D-QSAR protocol to predict the activity of enzymes and b) includes enzyme variants for model building^24, 25^.

The objective of this work was to apply QSAR principles to predict the kinetic properties of enzymes and obtain focused libraries to derive enzymes with desired activity. In a typical QSAR study different features of the small molecules such as molecular counts, molecular weight, topological features (2D-QSAR) or energy grid descriptors (3D-QSAR) are computed using different algorithms and these features are used to build statistical models that correlate with the observed drug activities^26–30^. As an evolution of 3D QSAR, Hopfinger and co-workers proposed 4D-QSAR method^31, 32^. The main difference is that the 4D-QSAR approach utilises the conformational flexibility of the ligand alone (Receptor Independent (RI)) or receptor complexed with ligand (Receptor Dependent (RD)) using methods such as molecular dynamics (MD). The generated ensembles are aligned and placed in a cubic grid where at each cell the occupancy measures are computed for the atoms of the aligned molecules and this is called as the grid cell occupancy descriptors, GCODs. The GCODs are generated for a number of different atom types (polar positive, polar negative, aromatic, hydrogen bond acceptor, hydrogen bond donor), called interaction pharmacophore elements, IPE. In a regular 4D-QSAR protocol the variations in biological responses are related to differences in the Boltzmann average spatial distribution of molecular shape with respect to the IPE^33^. The 4D-QSAR method has been successfully applied to design enzyme inhibitors of different drug targets, such as HIV-1 protease, HIV-1 integrase^34, 35^, p38-mitogen-activated protein kinase (p38-MAPK)^36^ and many others^37, 38^.

**Figure 1:**
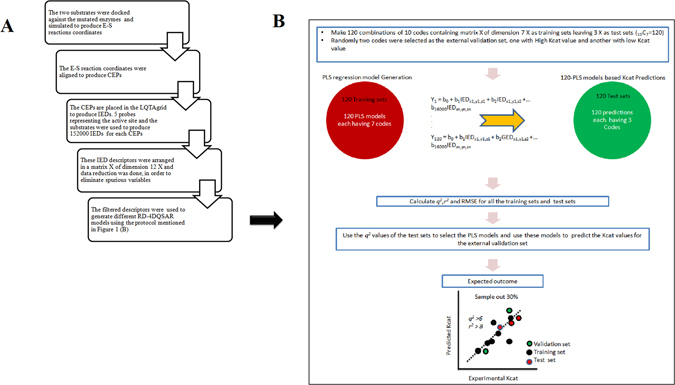
Flowchart of the overall RD-4D-QSAR process. (**A**) The chart explains the steps involved in the generation of interaction energy descriptors of the RD-4D-QSAR paradigm. (**B**) Schematic representation of the protocol that was used to generate different PLS models to derive models with maximum accuracy. Codes represent enzyme variants with different experimental *K*_*cat*_ values against two different substrates.

In this study we performed a receptor-dependent 4D-QSAR analysis on the variants of a serine protease that was observed with different enzymatic activities against two different substrates. We used the LQTA-QSAR method (LQTA, Laborato´rio de QuimiometriaTeo´rica e Aplicada) because it calculates intermolecular interaction energies at each grid point considering probes and all aligned conformations resulting from MD simulations^39^. The flow chart of the protocol is given in Fig. 1. LQTA is a new 4D-QSAR approach that starts with the generation of conformational ensemble profile, CEP, for each compound using MD simulation & alignment and these CEPs are used to generate the 3D descriptors. The unique feature of this methodology is that it explores jointly the main features of CoMFA (Comparative Molecular Field Analysis) and 4D-QSAR paradigms. The CEPs′ are placed in LQTAgrid defining a grid size to enclose all the atoms of the CEPs and a grid spacing of 1 Å, to generate several thousand points at the intersections of a regular 3D lattice. Different types of atoms called probes are used to compute the energy values of the interactions in a specific position of the grid. The energy values of selected probes at each grid point are called as interaction energy descriptors (IEDs). IEDs are the electrostatic and steric 3D properties computed for each grid point, based on the Coulombic and Lennard-Jones potential functions, respectively^39^. As usual the IEDs are arranged in a matrix and this matrix is used in a multivariate regression analysis wherein the biological activity is used as the dependent variable to construct the QSAR model.

Serine proteases are the most abundant and functionally diverse group among proteases. Bacterial protease subtilisin will cleave essentially any substrate, while another protease in the clotting cascade, Factor Xa, requires a four residue recognition sequence, Ile-Glu-Gly-Arg, in order to uniquely hydrolyse its polypeptide substrate after Arg^40, 41^. The molecular details of the catalytic mechanism involving these enzymes are well understood^42, 43^. New investigations on a number of serine proteases have changed our understanding of its function, regulation, and specificity^44^. Most of the serine proteases have three catalytic residues in the active site called the catalytic triad; Ser195, His57, and Asp102. These residues are conserved in all of the serine proteases, and are superimposable in the structures of these proteins^45^. To test the power of RD-4D QSAR methods we chose a serine protease that shows clear difference in the activity when a single position is substituted with different amino acids. Gly193, a key residue of serine proteases is highly conserved and when mutated shows significant variations in the catalytic activity of blood coagulation Factor XIa (FXIa)^46^. Gly193 is a part of type II *β*-turn which helps in the formation of oxyanion binding site and helps in retaining the S2′ site in the open conformation for binding of the P2′ residue of the substrates^47^. Non-Gly substitutions at 193 in FXIa causes reorientation in the peptide bond between amino acids 192 and 193, causing the amide nitrogen of residue 193 to point away from oxyanion binding site. Substituting Gly193 with Glu, Ala, Arg or Val shows distinguishable changes in the activity of FXIa due to modulation in the substrate binding modes. Enzyme studies with substrates S-2288 & S-2366, inhibitors diisopropylfluorphosphate (DFP) and *p*-aminobenzamidine (pAB) shows that the activity of the enzyme is most affected in FXIa_G193D_ & FXIa_G193V_, to a lesser extent in FXIa_G193E_ & FXIa_G193K_ and least impaired in FXIa_G193A_^47^. The objective of this work is to use RD-4D-QSAR method to accurately predict the kinetic properties of an enzyme specifically when the enzyme is mutated. A single mutation is preferred to measure the sensitivity of the QSAR models. Since, the functional role of position 193 is clearly demonstrated and mutations at this position show varying enzyme activity it was used as a case study to test our hypothesis. The FXIa activity values of 5 different substitutions at position 193 against two synthetic tripeptide substrates were used as the dataset to generate RD-4D-QSAR models. Our work is the first of its kind to demonstrate the predictability of a RD-4D-QSAR approach on a protocol where the enzyme/protein is mutated, as opposed to the standard practice where the varying component is the Ligand. This approach would demonstrate a faster and more accurate alternative to traditional screening methods and to obtain focused libraries with better enzymes.

## Experimental

### Data set, Docking and Molecular dynamics studies

Scheme A and B illustrates the customised RD-4D-QSAR protocol that was used in this study to predict the kinetic properties of mutant enzymes (Fig. 1). 12 reported enzyme assays of a protease enzyme (FXIa) pertaining to 5 different amino acid substitutions at position 193 against two substrates, H-D-Ile-Pro-Arg-p-nitroanilide (S-2288) and pyroGlu-Pro-Arg-p-nitroanilide (S-2366) were used as the dataset to generate the QSAR model^47^. The assays are alphabetically coded as given in Table 1 and henceforth will be referred using these codes. The enzymes activities were classified as low, moderate and high by identifying noticeable differences between the *K*_*ca*t_ values. To do this the *K*_*cat*_ values were sorted in the ascending order and the differences between the *K*_*cat*_ values were identified. Enzymes with *K*_*cat*_ < 40/sec showed an average difference of 10/sec between each other which were classified as enzymes with low activity. The next *K*_*cat*_ value above 40.8/sec is 64.8/sec which is 24/sec higher and since 64.8/sec and 71.2/sec falls closer to the average (69.3/sec) of all the 12 *K*_*cat*_ values, these were considered as enzymes with moderate activity. 98/sec is 26.8/sec higher than 71.2/sec and since till 117/sec the average difference between the activities were 6.3/sec, these were considered as enzymes with high activity. Finally, 145/sec is 28/sec higher than 117/sec which was considered as enzyme with very high activity.

**Table 1:** Table shows the experimental activity values of enzymes with different substitutions at position 193 against two different substrates.

Substitution at position 193	Substrate	Code	Kcat values	Classification
A	S-2288	A	64.8	Moderate
D	S-2288	B	4.2	Low
E	S-2288	C	71.2	Moderate
K	S-2288	D	30	Low
V	S-2288	E	10	Low
G (Wild)	S-2288	F	110.2	High
A	S-2366	G	117	High
D	S-2366	H	40.8	Low
E	S-2366	I	98	High
K	S-2366	J	114.2	High
V	S-2366	K	26.7	Low
G (Wild)	S-2366	L	145	very high

The enzymes are classified based on the *K*_*cat*_ values as explained in the text.

Swiss model server was used to generate different models of the protease enzyme containing mutations, Ala, Asp, Glu, Lys, and Val at position 193 using the crystal structure of FXIa as the template (PDB ID:1XX9). The reason for using this structure as the template was that the ligand binding mode in this structure reveals a substrate-like interaction in the active site of FXIa^48^. The generated models and the structure of human FXIa were used for docking the two substrates S-2288 and S-2366 using the program FlexX (BioSolveIT 2.0.2, 2011). Docking studies were conducted considering residues within radius of 6.0 Å of the active site to enclose the catalytic triad and the oxyanion hole. The docked conformations were selected based on the atomic distances between Ser195 and His57. The distances between the OH atom of Ser195 & carbonyl carbon of the substrate and the nitrogen (N1) of His57 & the amide nitrogen of the peptide bond between Arg & nitroanilide of the substrate were measured to select the substrate binding modes. The docked complexes were solvated by water molecules with orthorhombic cell shape of the explicit periodic boundary model. The solvated system was gently minimized with steepest descent algorithm until the tolerance reached 0.1 kcal/mol·Å, and further minimized by conjugate gradient algorithm until the tolerance reached 0.0001 kcal/mol·Å. The minimized system was gradually heated to 300 K, followed by the equilibration step for 300 ps. Finally, the production phase was carried out for 1000 pico seconds using an NPT ensemble at 300 K. During the MD simulations, the integration time step of 1 fs was used, and the SHAKE constraints were applied. MD simulations were performed by the CHARMm program, implemented in Discovery Studio v.3.1, with CHARMm force field version c35b5 and cff partial charges.

### Generating conformational ensemble profile (CEP) of the E-S complexes to compute interaction energy descriptors (IEDs) using LQTAgrid

As described above MD simulations of different E-S complexes were used to extract the E-S conformation for generating CEP. 1000 conformations of the enzyme active site complexed with the substrate; each conformation pertaining to 1 ps of the MD simulation were structurally aligned using the program do_multiprot^49^. The aligned E-S reaction coordinates are called as CEP. The CEPs of the 6 enzyme variants including the wild type were inserted into the LQTAgrid^39^ module to generate the interaction energy descriptors, IEDs. IEDs are calculated using the electrostatic and steric 3D properties for each individual grid point, based on the Coulombic and Lennard-Jones potential functions used by LQTA-QSAR method^39^. These descriptors are the interaction energies with the probe obtained from every conformation divided by the number of conformations. Each descriptor (IEDs) is labelled as “x, y, z_P_K” which represent the cartesian coordinates position of the selected grid cell (x, y, z) and the respective probe atom type (P) and “K” represents kind of interaction, Lennard-Jones interactions (LJ) or Coulombic interactions (C). A box size of 24 Å × 24 Å × 24 Å, with 1 Å resolution was used to compute interaction energy values for each IEDs. The probes used in this study were O-H, Ar (NH), SH, COO-, H_2_O which represents Ser, His, Asp, Met and water molecules in the active site of the enzyme. Each probe would generate 31250 grid points having x, y, z coordinate for the specified grid size and the grid spacing mentioned above and in each grid point LQTAgrid stores the LJ and C energies computed for the CEP’s of the 12 E-S reaction coordinates. Therefore, for 5 probes 156,250 IEDs were generated and these descriptors were arranged in a matrix X of dimension 12X. A data reduction was done, in order to eliminate spurious variables^50^.

### Variable selection and QSAR model generation

The energy cut-off of Lennard-Jones and Coulomb descriptors was carried out using the formula mentioned elsewhere^51^. The energy values ≥30 kcal/mol (125.52 kJ) for Lennard-Jones descriptor or Coulomb descriptors computed at an x, y, z position were filtered by taking the logarithmic value of the residual and adding this value to 30 kcal/mol.

$$\begin{array}{c}L{J}_{x,y,z}or\,Co{l}_{x,y,z} < 30\,kcal/mol=LJ\,or\,Col\\ L{J}_{x,y,z}or\,Co{l}_{x,y,z}\ge 30\,kcal/mol=LJ^{\prime} \,or\,Col^{\prime} =30+log(LJ^{\prime} \,or\,Col^{\prime} /\,kcal/mol-30)\end{array}$$

Next level elimination was done on the IEDs that had an absolute individual correlation coefficient (*r)*, with the activity values <0.5. Correlation analysis between the IEDs and dependent variable (*K*_*cat*_ values) was conducted for the codes of the training set and the test set containing 10 codes leaving the external validation set. The training sets contained 7 codes, the test sets contained 3 codes and external validation set contained two codes, one with high *K*_*cat*_ value (Code K = 117/sec) and another with low *K*_*cat*_ value (Code D = 30/sec). These two activities were selected as external validation set to test the efficiency of the QSAR models generated in this study to differentiate enzyme variants with high and low activity. To generate the Partial least squares regression (PLSR) models the dataset was divided into training set and test set. The selected IEDs of the 10 codes derived from each E-S CEPs were used to generate QSAR models. The IEDs of the 10 codes were arranged in a matrix X of dimension 10X and this dataset was used to derive different combinations of training sets and test sets. 120 RD-4D-QSAR models were generated in this study to filter models with reasonable QSAR statistics. To generate a QSAR model 10 codes were split into two, 7 codes as the training set and the remaining 3 codes as the respective test set. Like this 120 sets containing 7 codes in each set were used as the training sets and 120 sets containing 3 codes in each set were used as test sets (_10_C_3_ = 120). The PLS regression model derived from the training sets were used to predict the *K*_*cat*_ values for the test sets. All the 120 training sets were used to generate the PLS models and each model was validated applying the leave-one-out (LOO) cross validation method using PLSR package version 2.3-0 of the Revolution R statistical tool^52^. *K*_*cat*_ values of the respective codes mentioned in Table 1 was used as dependable variable (Y) and the selected IEDs were used as the independent variables (X) to generate the PLS models. The 120 PLS models were used to predict the *K*_*cat*_ values of all the codes that were present in the respective 120 test sets. Validation of QSAR models were done by calculating *q* ^2^, *r* ^2^ and root mean square error (RMSE) values for all the training sets and test sets. As mentioned above the external validation is the only way to establish a reliable QSAR model^53^. Therefore, the models showing a *q* ^2^ value > 0.4 for the test sets were used to predict the *K*_*cat*_ values for the external validation set. Finally, visualisation studies of the IEDs derived from the best PLS model were illustrated in the 3D space employing DS Visualizer software^54^ and a detailed interpretation was provided to show crucial atomic details of the E-S interactions that is important for enzyme activity.

### Sensitivity and specificity tests

Few assumptions were made to differentiate FXIa enzymes with high, moderate and low activity (Table 1). To differentiate high and low activity the middle value 52/sec (between 40/ sec and 64/sec) and 85/sec (between 71.2/sec and 98/sec) were chosen. Enzymes with *K*_*cat*_ value <52/sec were considered as enzymes with low activity and enzymes with *K*_*cat*_ value > 85/sec were considered as enzymes with high activity. Different limits were defined to assign true positives (TP), true negatives (TN), false positives (FP) and false negatives (FN). (TN) <85> (TP) code represents enzymes showing both experimental and predicted *K*_*cat*_ values <85 as TN and >85 as TP. The mismatches of the same, wherein the predicted value is >85 and the corresponding experimental value is <85, represents FP and vice versa as FN. (TP) <52> (TN) code represents enzymes showing both experimental and predicted *K*_*cat*_ values <52 as TP and >52 as TN. The mismatches of the same, wherein the predicted value is >52, while the corresponding experimental value is <52, represents FN and vice versa as FP.

## Results

### Docking and simulation studies

Docking studies were conducted to obtain catalytic binding modes of the substrates S-2288 and S-2366 in the modeled structures of 5 mutant enzymes and the wild type enzyme. The docking conformations were selected based on the catalytic binding mode of the amide bond of the substrates in the active site of the serine protease (Fig. S1, Supplementary Material). In the selected conformations the carbonyl group of the hydrolyzing amide bond and the amide nitrogen of the substrates were close to Ser195 and His57 respectively, giving a plausible model of Michaelis complexes for MD simulations^55^. The docking energies of the substrates range from −49.7 kcal/mol to −8.17 kcal/mol (Table S1, Supplementary Material). There were no correlation between the *K*_*cat*_ values of the enzymes and best docked energies neither did the docked energies of the selected conformations showed any correlation with the activity. Therefore, the selected docked conformations were considered for simulation studies and the binding energies were discarded. The docked E-S complexes show small variations in binding mode, i.e. the substrates’ conformations in the binding pocket of the mutant and the wild type enzymes were slightly different from each other. Post simulation studies show a “T” conformation of the substrates in the active site of the enzyme wherein the side chains of the substrates occupied 3 different cavities. H-D-Ile-Pro occupied pocket 1 where it shows charge based interactions with Asp189 and Gly216. Arginine of the substrate occupied pocket 2 where it shows charge based interaction with Glu96 and cation Pi interactions with His51. p-nitrolinamide occupied pocket 3 where it shows strong charged based interaction with Arg33. The amide bond of the substrates between Arginine and nitrolinamide of the substrates lies in the center buried between the Ser195 and His57 (Fig. S2A and B, Supplementary Material) and it is surrounded by water molecules that are important for hydrolysis (Fig. S2C, Supplementary Material). The simulations of the active site complexed with the substrate showed stable interactions. Similar interaction energy values were observed for simulations of the whole enzyme-substrate complex and the partial enzyme-substrate complex (Fig. S3). The calculations were carried out using *g_mmpbsa* tool. One of the objectives of the study was to use this protocol as a screening tool for enzyme engineering. Therefore, using the partial enzyme for simulations would drastically reduce the time for screening variants. In this case the enzyme’s active site is present in the surface and the substrate is exposed to the solvent molecules (Fig. S2). Residues of the enzyme within 6.0 Å radius of the substrate completely cover the substrate as it is in the actual enzyme- substrate complex. Moreover, since the simulation was conducted for a short period (1 nanosecond) it did not affect the structure of the enzyme. 6.0 Å radius is not universal and it should be customized for different enzymes i.e. for buried active sites the selections should be made appropriately.

### QSAR model generation

**Figure 2:**
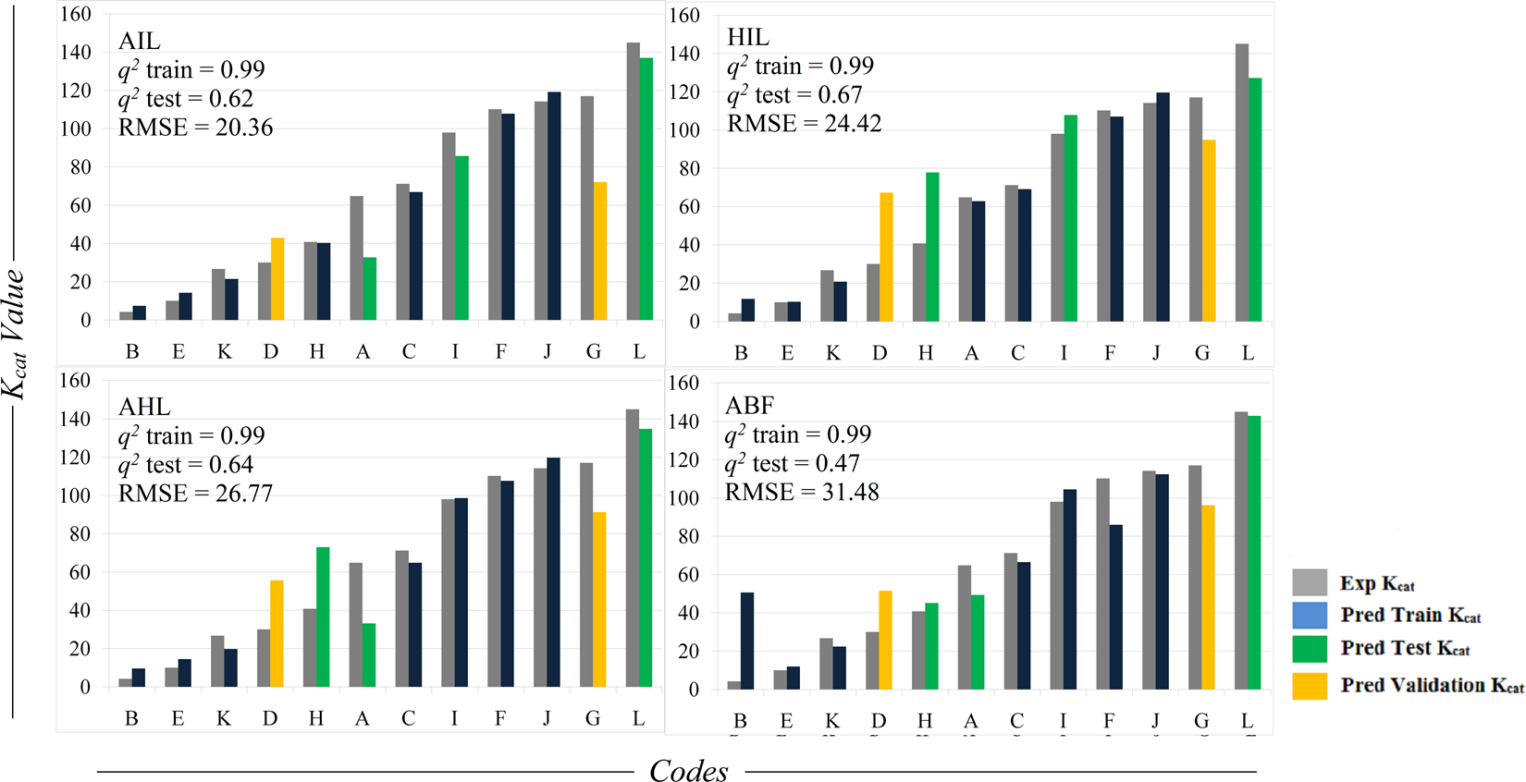
Analysis of the predicted activity values generated using PLS models derived from 1875 IEDs chosen based on the *r* value > 0.5. Graph shows the experimental vs. predicted activity values; training sets containing 7 codes (blue), test sets containing 3 codes (green); external validation set containing 2 codes (orange) and the respective experimental values (grey). The models sorted based on the least RMSD values of the training and the test set were used to predict the activity of the validation set (Table S5). The predictions on the validation set showed clear difference between the enzyme variants with high and low activity; however, the predicted *K*_*cat*_ values were only a little closer to the experimental values.

**Figure 3:**
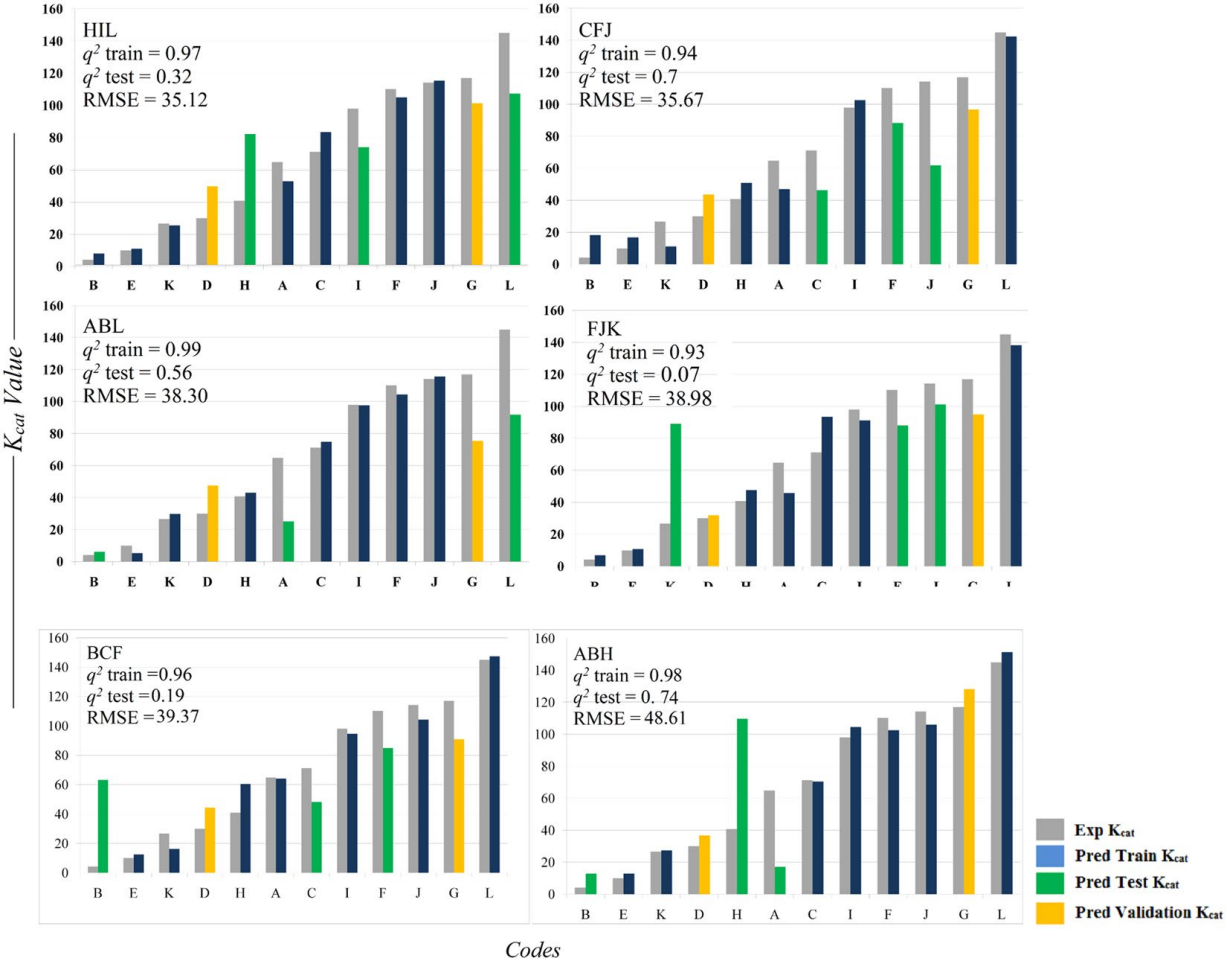
Analysis of the predicted activity values generated using PLS models derived from 6188 IEDs chosen based on the *r* value > 0.45. Graph shows the experimental vs. predicted activity values; training sets containing 7 codes (blue), test sets containing 3 codes (green); external validation set containing 2 codes (orange) and the respective experimental values (grey). The models sorted based on the least RMSD values of the training and the test set were used to predict the activity of the validation set (Table S6). The predicted *K*_*cat*_ values were very much closer to the experimental values.

**Figure 4:**
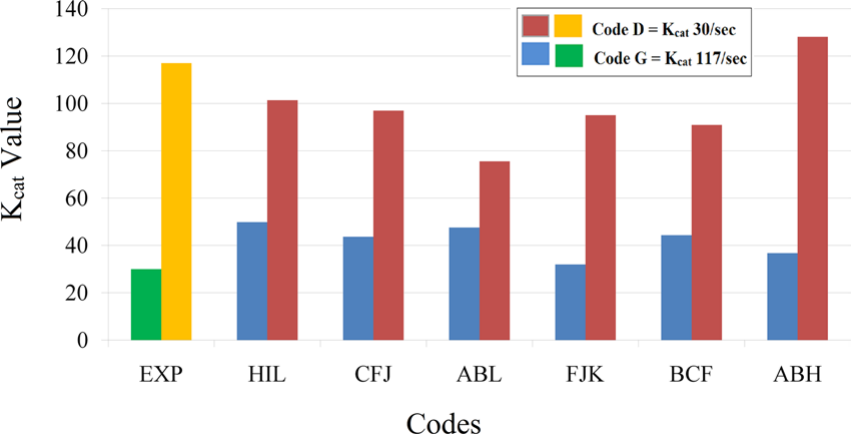
Graph of experimental vs. predicted activity of the validation sets of the models derived from 6188 IEDs chosen based on the *r* value > 0.45.

The protocol used for generating 4D QSAR models is given in Fig. 1. LQTAgrid generated 156,250 IEDs using 5 different probe atoms and CEP derived from E-S simulation of each mutation. The probe explores every grid point of a 1 Å grid cell lattice and 3D energy interaction descriptors were calculated at each grid point for every CEP. 156,250 IEDs were arranged in a matrix X of dimension 12X for the 12 codes totally summing up to1875000 IEDs (Table S2, Supplementary Material). 10% of 156,250 IEDs were observed with zero energy values. Correlation analysis between the IEDs and dependent variable (*K*_*cat*_ values) was conducted for the codes of the training set and the test set (10 codes) leaving the external validation set. Correlation analysis between the IEDs and the dependent variable (*K*_*ca*t_ values) showed 1120 descriptors with zero *r* values. The remaining 139,505 IEDs were used to obtain IEDs that showed an *r* value ≥ 0.40 against the *K*_*cat*_ values. Three sets of IEDs were chosen for generating the regression models; 1875 IEDs that showed *r* value ≥ 0.5, 6198 IEDs with *r* value ≥ 0.45 and 19,764 IEDs with *r* value ≥ 0.4 (Tables S3 and S4, Supplementary Material). Using a systematic approach by including 7 codes in the training set, 120 PLS models were produced. These models were used to predict the *K*_*cat*_ values of 120 test sets containing 3 codes each. Initially, 1875 IEDs were chosen based on the *r* value > 0.5 to generate the 4D QSAR models. 18 out of 120 models showed *q* ^*2*^ > 0.3 for test sets. Of these the top 5 models sorted based on the RMSE value were used to predict the activity values for the validation set. The predictions on the validation set showed clear difference between the enzyme variants with high and low activity; however, the predicted *K*_*cat*_ values were only a little closer to the experimental values. The predicted low *K*_*cat*_ values of the top 3 out of these 5 models were below 52/sec which was the same as its experimental activity. Similarly, the predicted high *K*_*cat*_ values of these models were above 85/sec which was also the same as its experimental activity. The noticeable point is that, none of the enzyme variants with low activity were predicted as variants with higher activity and vice versa (Fig. 2 and Table S5, Supplementary Material). A more judicious choice of parameters would probably permit a closer fit. The accuracy of the 120 models improved on using 6188 descriptors with *r* value > 0.45. 9 out of these 120 models showed *q* ^*2*^ > 0.1 (7.5%), especially the top 5 models showed *q* ^*2*^ > 0.25 revealing correlation between the predicted and the experimental activity values. The top 9 models were sorted in the ascending order of the RMSE values and the models with RMSE < 40 were used to predict the activity of the validation set (Table S6). Models HIL, CFJ, ABL, FJK, BCF and ABH were used to predict the activity of the validation set (Fig. 3). The intercepts and PLS coefficients of these models are given in Excel sheets (Tables S7–S12 respectively). 3 of 4 models clearly differentiated enzyme variants with high activity against the variants with low activity. Apparently, the difference in the *K*_*cat*_ value was > 50 between the enzymes showing high and low activity. Impressive results were observed for the model CFJ were the experimental *K*_*cat*_ values 30.0/sec and 117.0/sec for two different mutations at position 193 of the enzyme were predicted to be 43.6/sec and 97.0/sec respectively (Fig. 4).

**Figure 5:**
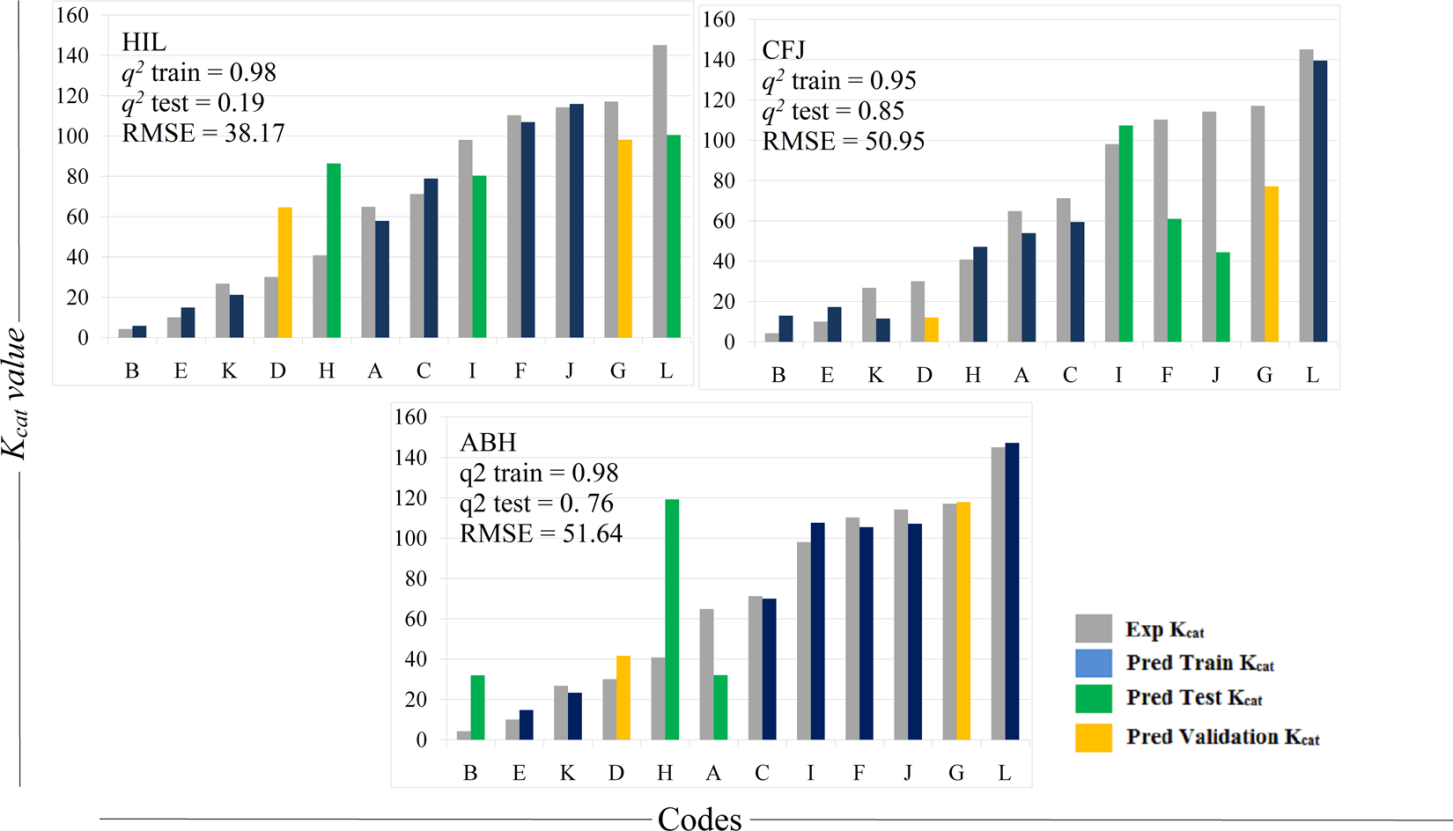
Analysis of the predicted activity values generated using PLS models derived from 19764 IEDs chosen based on the *r* value > 0.4. Graph shows the experimental vs. predicted activity values; training sets containing 7 codes (blue), test sets containing 3 codes (green); external validation set containing 2 codes (orange) and the respective experimental values (grey). The models sorted based on the least RMSD values of the training and the test set were used to predict the activity of the validation set (Table S13). These models showed impressive *q2* values but the RMSE values of the test set were relative higher than that of the previously obtained models.

The models were vigilantly chosen to avoid false positives. To demonstrate this we have chosen two models one with high *q* ^*2*^ value & high RMSE (model ID: ABH) and the other with low RMSE & low *q* ^*2*^ value (model ID: FJK). In both cases, enzyme variants in the test sets with low experimental activity were predicted as enzymes with high activity. Therefore, even though the activities derived using these models for the validation set seems to match with the experimental values it might lead to false positive predictions for some other mutations (Fig. 4, Tables S11 and S12). 19764 IEDs were obtained by further scaling down the *r* value to 0.4. The models generated using 19764 IEDs show impressive *q* ^*2*^ values but the RMSE values of the test set were relative higher than that of the previously obtained models. The model showing the least RMSE value (HIL) predicted the enzyme variants with high activity correctly; but it predicted variants with low activity as enzymes with moderate activity (Table S13). The model with *q* ^*2*^ 0.77 (ABH) showed an impressive prediction for the validation set i.e., the experimental *K*_*cat*_ values 30.0/sec and 117.0/ sec for two different mutations at position 193 of the enzyme were predicted to be 41.6/sec and 177.8 /sec respectively (Fig. 5.). From this it can be concluded that models generated using 6188 descriptors with *r* value > 0.45 gave best results.

### IEDs shows atomic interactions that are crucial for enzyme activity and specificity

The correlation coefficients of the IEDs against the enzyme activity and PLS regression coefficients (*rc*) of the best model were used to locate IEDs on different CEPs to identify residues that affected or enhanced the activity of the enzyme. Correlation studies between IEDs and activity revealed that there were more number of IEDs with negative *r* values than positive *ones* in the active site*. N*egative *r* values state that the activity values of the codes increases as the energy values stored in different IEDs decreases. Lower energy values suggest stable intermolecular interactions. To explain this, a graph is plotted with a few LJ descriptors and the activity values of the 12 codes (Fig. S4, Supplementary Material). The graph shows that as the LJ energy values decrease the activity of the codes increases. There were 240 IEDs with negative *r* value (<−0.60) and of these, 179 descriptors were “LJ” IEDs and 61 were ‘C’ IEDs. The blue mesh shown in Supplementary Fig. S5 represents IEDs with negative correlation values observed within 2 Å radius of the active site. The residues falling in this region were Cys191, Gly218, Cys219, Gly193 and Asp194. It can be concluded that reducing the intermolecular energy values between the enzyme and substrates by mutating some of these residues in the active site could improve the activity of the enzyme towards S-2288 and S-2366.

**Figure 6:**
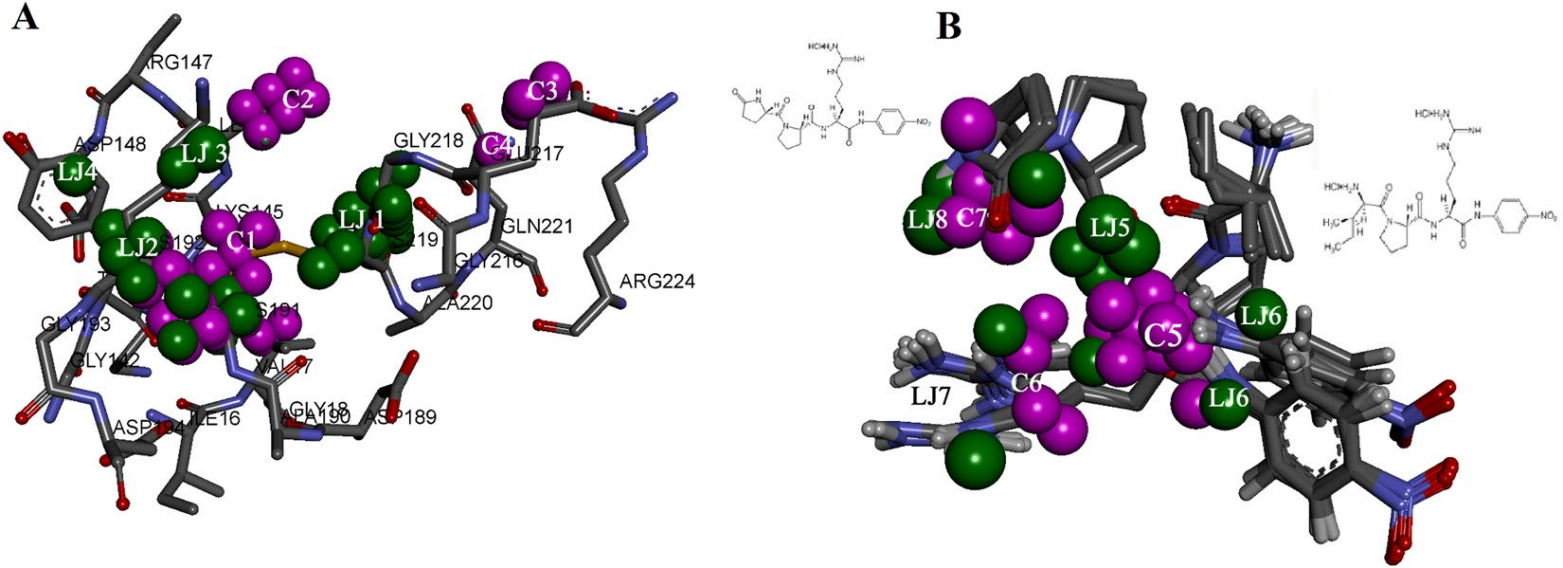
The IEDs of the best QSAR model with negative *rc* that are crucial for enzyme activity were mapped on the active site of the enzyme to locate important E-S interactions. (**A**) Represents the LJ (green) and C (pink) IEDs on the CEPs of active site of the enzymes and (**B**) represents the same on the substrates. The 2D structures of the substrate S-2366 (left) and S-2288 (right) represent the binding conformations of pyroGlu-Pro and H-D-Ile-Pro of the substrates in the active site. This conformational change is one of the crucial differences observed in the active site that defines the specificity of the enzymes.

**Figure 7:**
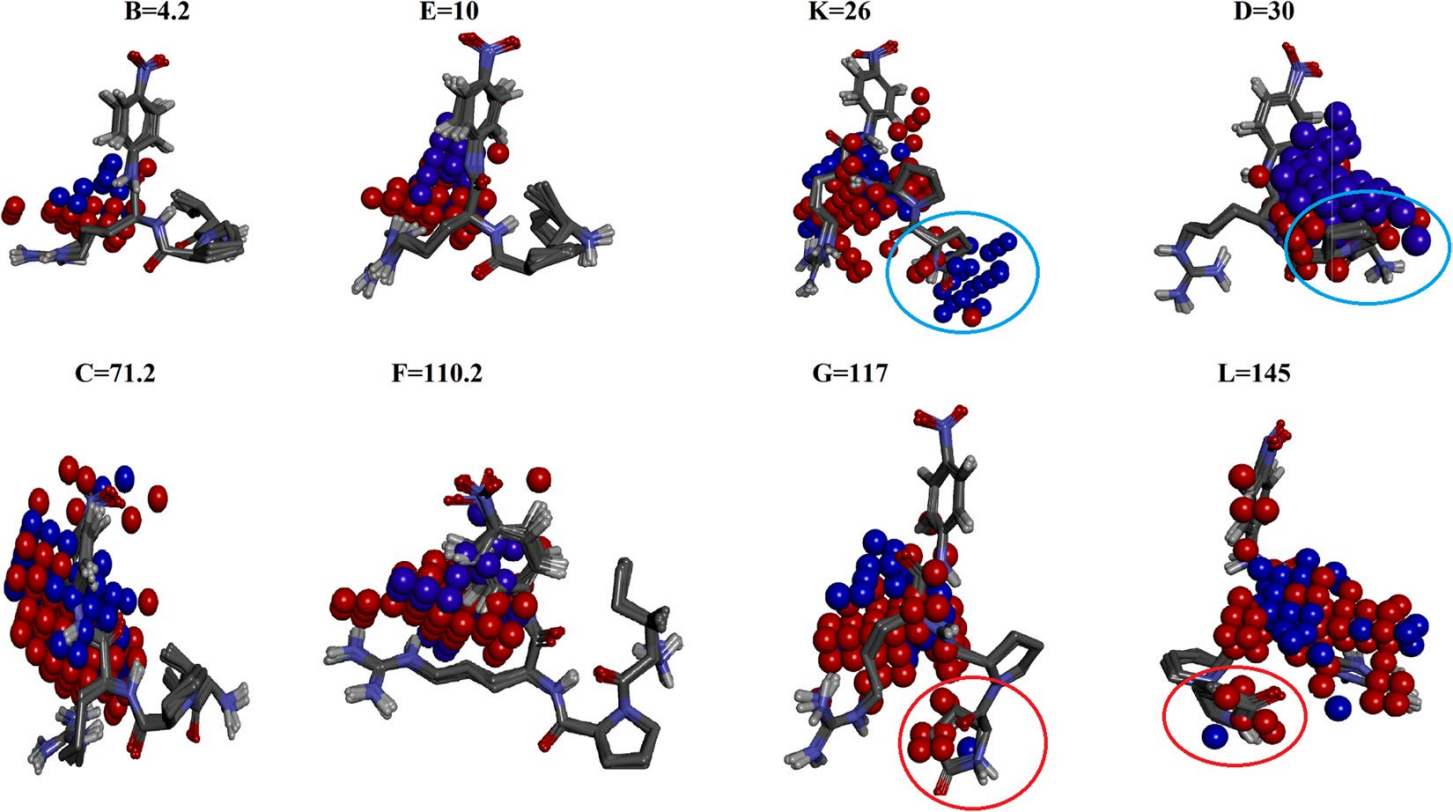
IEDs with positive and negative *rc* derived from the best QSAR model mapped on the substrates. Clear differences are seen in the arrangement of IEDs, precisely correlating with the enzymes showing high and low activity. The blue and red spheres represent IEDs with positive and negative *rc* respectively, within 2.5 Å radius to the substrate conformations. The alphabets represent the codes and the respective substrate conformations in the CEPs of a specific enzyme variant. The number represents the *K*_*cat*_ values. The regions encircled in blue and red over pyroGlu moiety are IEDs that differentiated enzymes with low and high activity respectively.

The *rc* of the IEDs of the of the model code HIL that gave the best prediction for the external validation set were graphically mapped on the active site residues and the substrates to show the important E-S interactions and its effect on enzyme activity. This was done by comparing the IEDs with negative *rc* (red) & positive *rc* (blue) on the CEPs of the substrates and the corresponding LJ & C regions on the CEPs of the E-S. The IEDs with negative *rc* were mapped on the active site of the enzyme and the substrates and it was appropriately labelled as LJ and C regions. The negative regression coefficients of IEDs suggest that conformers with low energy content are the important ones for the enzyme activity. There were six LJ descriptor regions (green) and seven C descriptor regions (pink) spotted on the aminoacids in the active site and on the substrates that influenced the activity of the enzyme (Fig. 6). The first four LJ and C descriptor regions (Fig. 6A) are the probes on the active site residues that store the energy potentials of the interactions with the substrates. The rest of the IEDs (Fig. 6B) are probes closer to the substrates’ conformations. The interactions of nitroanilide of the substrate with Gly193 and Lys192 are stored in LJ2 and LJ3 descriptor regions respectively. The potentials for π-π interaction between the substrates and Tyr143 are stored in LJ4. C1 descriptor region is over the peptide linkage between arginine and nitroanilide which stores the potentials that is important for the orientation of the peptide bond hydrolyzed by the enzyme. C1 and C5 regions close to His57 and Ser195 stores the potentials of the; interaction of water, serine, histidine and the amide bond involved in hydrolysis. The LJ1 descriptor region mapped on the backbone of Cys219 and Ala190 may be involved in the hydrophobic interaction with the side chain carbons of Arg of the substrates. LJ7 region on Asp189 contains the potentials for charge based interactions with the side chain of Arg of the substrates. LJ2 and LJ3 regions are close to Gly193 and side chain of Lys192. These two residues stabilize the binding conformation of the amide bond between Arg and p-nitroanilide of the substrates to form a proper attack conformation, placing it correctly towards Ser195 and His57 for hydrolysis. C1 descriptor region was found on the back bone of Cys191, Cys219 and some side chain carbons of Arg of the substrates, storing the potentials for hydrophobic interactions. LJ1 region on Gly218, C3 region on Glu217 and LJ8 & C7 regions on pyroGlu-Pro of S-2366 stored potentials specific to S-2366, the substrate for which the enzyme shows higher hydrolysis rate. Therefore, residues close to LJ1, C3 and LJ8 & C7 regions can be used to design enzyme and substrates for faster hydrolysis. C2 descriptor region on Leu146 stores potential for hydrophobic interactions with the H-D-Ile moiety and pyroGlu moiety of the substrates. LJ5 descriptors region are closer to the carbonyl and the amide group of proline of the substrate. C5 forms very important descriptor region covering the peptide linkage between Arg and nitroanilide of the substrates that stores the potentials important for hydrolysis. LJ6 descriptor region was close to the ring of nitroanilide. C6 & LJ7 regions were close to the side chains of charged nitrogens of Arg of the substrate that stores electronegative potentials. Mapping IEDs on the substrates based on the *rc* revealed active site residues that showed specific interactions of the enzyme with the two substrates used in this study (Fig. 7). There were more IEDs with positive *rc* values close to substrate conformations extracted from the enzyme mutations showing low activity. These positive *rc* values are detrimental for the enzyme activity. This is depicted in Fig. 7 specifically CEPs presented in the first row encircled in blue. Moreover, these IEDs were concentrated near the amide bond between the Arg and p-nitroanilide. Conversely, there were more IEDs with negative *rc* values for the enzyme variants with moderate and high activity. Detailed visualization studies revealed that additional negative IEDs were present near the pyroGlu moiety of S-2366 for enzymes showing high activity, specifically for codes F, G and L (Fig. 7). In contrast, for enzymes with low activity (Eg: Code K in Fig. 7) this region was occupied with positive IEDs. Also, there were more of negative IEDs on the Arg of the substrates for enzymes showing high activity. As mentioned above negative *rc* of IEDs suggests that conformers with lower energy are the important ones for the enzyme activity. Therefore, the positive IED regions mapped on the substrates, specifically those derived from enzymes with low activity can be explored for designing inhibitors against serine proteases.

### Specificity of FXIa

The IEDs with positive *rc* and negative *rc* close to the substrates, specifically in codes with low activity B, D, & E and codes with high activity G & L (Fig. 7) that correspond to enzymes with mutations D, K & E and A & G at position 193 respectively, showed specific interactions with the substrates. IEDs mapped on the pyroGlu-Pro of S-2366 specific to codes G & L, labeled as LJ8:C7 in Fig. 6 shows specific interaction with Glu217 and Gly218 in the active site. Therefore, this specific site of the enzyme, explains the molecular basis for substrate specificity. Also, for the specific interaction the enzyme prefers amino acids with smaller side chain at position 193 because Code G and L had Ala and Gly at position193 respectively. On the other hand the slightly bulkier amino acid D, K and E in position 193 sterically affects the orientation of nitroanilide of substrate S-2288 which in turn shifted the H-D-Ile-Pro moiety of S-2288 away from Glu217 and Gly218. Therefore, E-S CEPs with high activity showed specific interactions with Glu217 and Gly218, this perhaps could be the site in FXIa that determines specificity of the enzyme.

### Effect of the mutations at 193 on enzyme activity

The orientation of Arg of the substrate with respect to the nitroanilide moiety was different in enzyme variants showing low activity and high activity. Arg of the substrates formed a linear conformation in codes F, G & L (high activity mutants) compared to the codes B, D, E & K (low activity mutants) that shows a bent conformation. For example, a comparison between mutation D at position 193 of the enzyme with least activity against S-2288 and mutation A at position 193 of the enzyme with the highest activity against S-2366 is shown in Supplementary Fig. S6. The CEPs of mutations A at position 193 complexed with S-2366 showed a prominent ‘T’ conformation where as the CEPs of mutation D with S-2288 showed an acute angle between the nitroanilide and Arg. Since there is a difference in the orientation of Arg between enzymes with higher and lower activity the IEDs on the Arg conformations were also different in these codes. Conformations of Arg of the substrates in Codes F, G and L were closer to LJ1 region that encloses Gly218. Apart from this the LJ1 region was also close to Gly226, Gly216 and the backbone of Trp215 & Thr213. Therefore, these residues could be predicted as the hotspots that can be used to increase the activity of the enzyme towards S-2366 and S-2288.

### Sensitivity and specificity tests

The models derived using 6188 descriptors with *r* value > 0.45 and RMSE lesser than 40 effectively differentiated enzymes showing high and low activity. However, these models were less efficient in predicting enzymes with moderate activity. Sensitivity test values of these models were above 50% for the different limits mentioned above. The values pertaining to the specificity of these models were above 80%. (Table 2). The external validations were successful, showing predicted activity values close to the experimental activity values. Moreover, the top ranked models of the training set, test set and the external validation set unambiguously differentiated enzymes with high and low activity.

**Table 2:** Specificity and sensitivity tests of the QSAR models. <85> and <52> are the limits used to differentiate enzymes with high and low activity which is clearly defined in the methods section. The complete calculations can be found in Table S14.

Limits	Sensitivity	Specificity
**Predicted (HIL)**		
**<85>**	0.8	1.0
**<52>**	0.8	1.0
**Predicted (CFJ)**		
**<85>**	0.8	1.0
**<52>**	1.0	0.7
**Predicted (ABL)**		
**<85>**	0.8	1.0
**<52>**	1.0	0.9
**Predicted (FJK)**		
**<85>**	1.0	0.8
**<52>**	1.0	0.9
**Predicted (BCF)**		
**<85>**	1.0	0.7
**<52>**	1.0	0.8
**Predicted (ABI)**		
**<85>**	1.0	0.8
**<52>**	0.9	1.0

## Discussion

Previously 3D-QSAR principle was used to predict the substrate specificity of different enzymes. Example, selectivity of Penicillin G Amidase was predicted using 3D-QSAR regression models which correlate *K*_*cat*_/*K*_*M*_ to the descriptors of the substrates^56^. In a study where the conformational factors responsible for the activity and substrate specificity of alkanesulfonate monooxygenase were identified, the PLS models show that the steric and electronic factors could reduce the activation energy of the rate determining step of the reaction^57^. Therefore, 3D-QSAR methods have been successfully used for predicting enzyme activity. For the first time, we have incorporated mutations in the enzyme structure and applied RD-4D-QSAR formalism and successfully predicted the enzyme activity (*K*_*cat*_) with high accuracy. The key reasons for the success were; (a) the enzyme-substrate complexes were simulated using molecular dynamics to produce molecular motion over time, a crucial step proven to be important for deriving a successful QSAR model^58^. (b) The use of a methodology that explores jointly the main features of CoMFA and 4D-QSAR paradigms^31^. (c) The use of 5 different probes to generate IEDs that represent the active site of the enzyme and the substrate of each E-S simulation. (d) Finally a systematic approach was used to derive the PLS models that include datasets of the mutated enzymes in the training set. RD-4D-QSAR models were able to capture the existence of specific induced-fit interactions between the enzyme active site and inhibitors^59^. It was also used to visualize spatial maps of atom types that are important on the comprehension of the enzyme-ligand interaction mechanism^60^. Similarly in this study we were able to specify the role of some active site interactions and its energetics in the catalytic efficiency of the enzyme. In our study the RD-4D-QSAR models generated for the mutations of FXIa showed similar changes in the enzyme activity as reported in the kinetic studies^38^. Also the predicted activity values showed major changes for mutations, FXIa_G193D_ and FXIa_G193V_, and minor changes for mutations FXIa_G193K_, FXIa_G193E_ and FXIa_G193A_. Post simulation analysis of the enzyme-substrate complex showed a ‘T’ conformation of the substrates in the active site of the enzyme, specifically in enzymes showing high activity, e.g., G193A (code G = 117/sec). A total of 1875000 data points (IEDs) were generated for 12 simulations of the E-S complexes. 9 out of 120 models showed a *q* ^*2*^> 0.1 for the test sets and these were used to predict the activity of the validation set. Predictions on the validation set showed an average *q* ^*2*^ of 0.8 ± 0.17 and RMSE of 16 ± 8.3 with a least RMSE of 8.6 for the validation set. This reveals that the predicted activity values are closer to the experimental activity values. Moreover, these models showed> 80% specificity and >50% sensitivity revealing that the top ranked models of the training set, test set and the external validation set unambiguously differentiated enzymes with high and low activity. For example, the experimental *K*_*cat*_ values 30.0/sec and 117.0/ sec for the two different mutations at position 193 of the enzyme were predicted to be 43.6/sec and 97.0/sec respectively. The 4D-QSAR descriptors were plotted on the E-S CEPs and specific active site residues were identified for enzyme specificity and enzyme activity. Gly218 & Glu217 were predicted to be involved in substrate specificity pertaining to the change in position 193 and Cys191, Ala190, Asp189 & Gly218 were predicted to be the crucial hotspots governing enzyme activity. Computational time for screening is an important factor that has to be considered where such high throughput screening is employed. The protocol takes ~5 minutes to derive the activity of 1 variant; therefore ~2000 variants can be screened in a week and this can be improved by increasing the number of processors. It took ~4 minutes to generate 1 nano second simulation for residues within 6 Å radius of the substrate (~9000 atoms including water molecule) with a computer containing 20 processing cores. It took ~1 min to generate IEDs and predict the activity using the regression model. Screening of ~2000 variants in a week is quite an achievement with the accuracy of the model mentioned in this paper.

## Conclusions

In an industry that is strained for improved performance of enzymes and has direct impact on the economics of production, our approach is a useful tool to shorten the evolutionary cycle for delivering enzymes of desired properties. We have designed a novel method that incorporates molecular motions of the enzymes into RD-4D-QSAR formalism to predict enzyme activity. This method that predicts enzyme activity with high accuracy can be used to screen enzyme modifications/mutations and derive focused library with high confidence level. The interaction energy descriptors of the best QSAR model mapped on the E-S CEPs were used to predict residues responsible for enzymatic activity and substrate specificity. These sites can be used as hotspots for designing proteases with better activity and specificity. Finally, this computational method showed reasonable computer performance which can be faster and less expensive than high-throughput screening of enzyme libraries.

## Electronic supplementary material

Supplementary Data

Table S1

Table S2

Table S3

Table S4

Table S5

Table S6

Table S7

Table S8

Table S9

Table S10

Table S11

Table S12

Table S13

Table S14
