# Supplementary material for: A receptor dependent-4D QSAR approach to predict the activity of mutated enzymes
Source: Sci Rep. 2017 Jul 24;7:6273. doi: 10.1038/s41598-017-06625-x (PMC5524700; doi:10.1038/s41598-017-06625-x)
Supplement: Supplementary file 1 — Supplementary Data [file 41598_2017_6625_MOESM1_ESM.pdf]

A receptor dependent-*4D QSAR* approach to predict the activity of mutated enzymes

R.Pravin Kumar[1] and Naveen Kulkarni[1]

Supplementary data

Supplementary Figures

Supplementary Figure 1

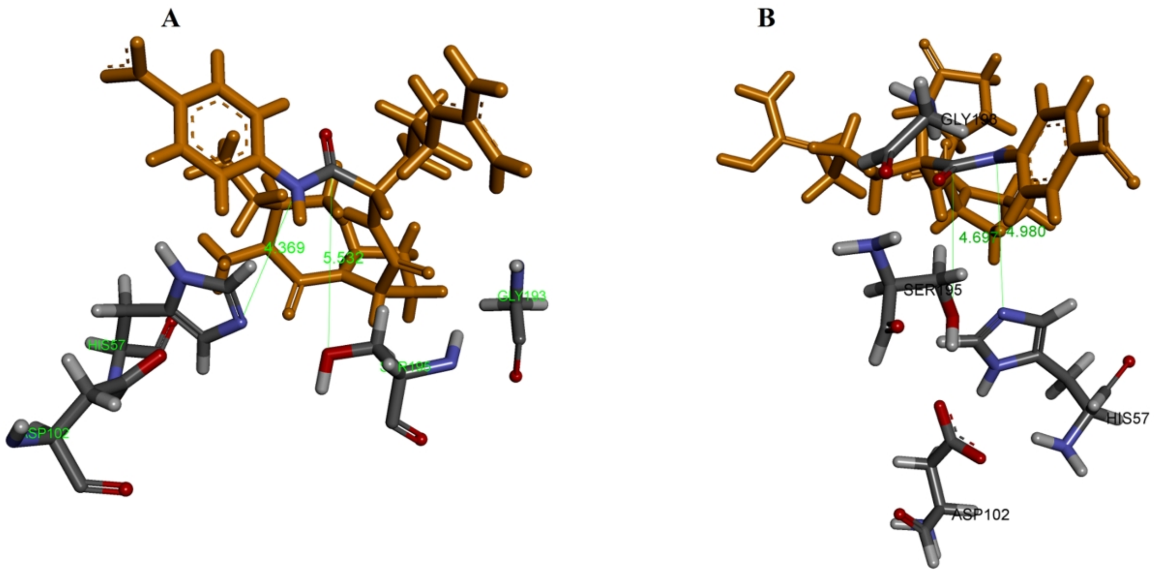

Fig. S1. Stick model of the docked conformation of the substrates (orange) in the active site (elemental model) of the wild type enzyme. A and B are the selected docked conformations of S-2288 and S-2366 in the active site of the enzyme. The green lines depict the distances (Å) between the atoms OH of Ser195 and carbonyl carbon of the substrate and the nitrogen (N1: more basic or double bonded) of His57 and the nitrogen of the amide bond between Arg and p-nitroanilide of the substrates.

Supplementary Figure 2

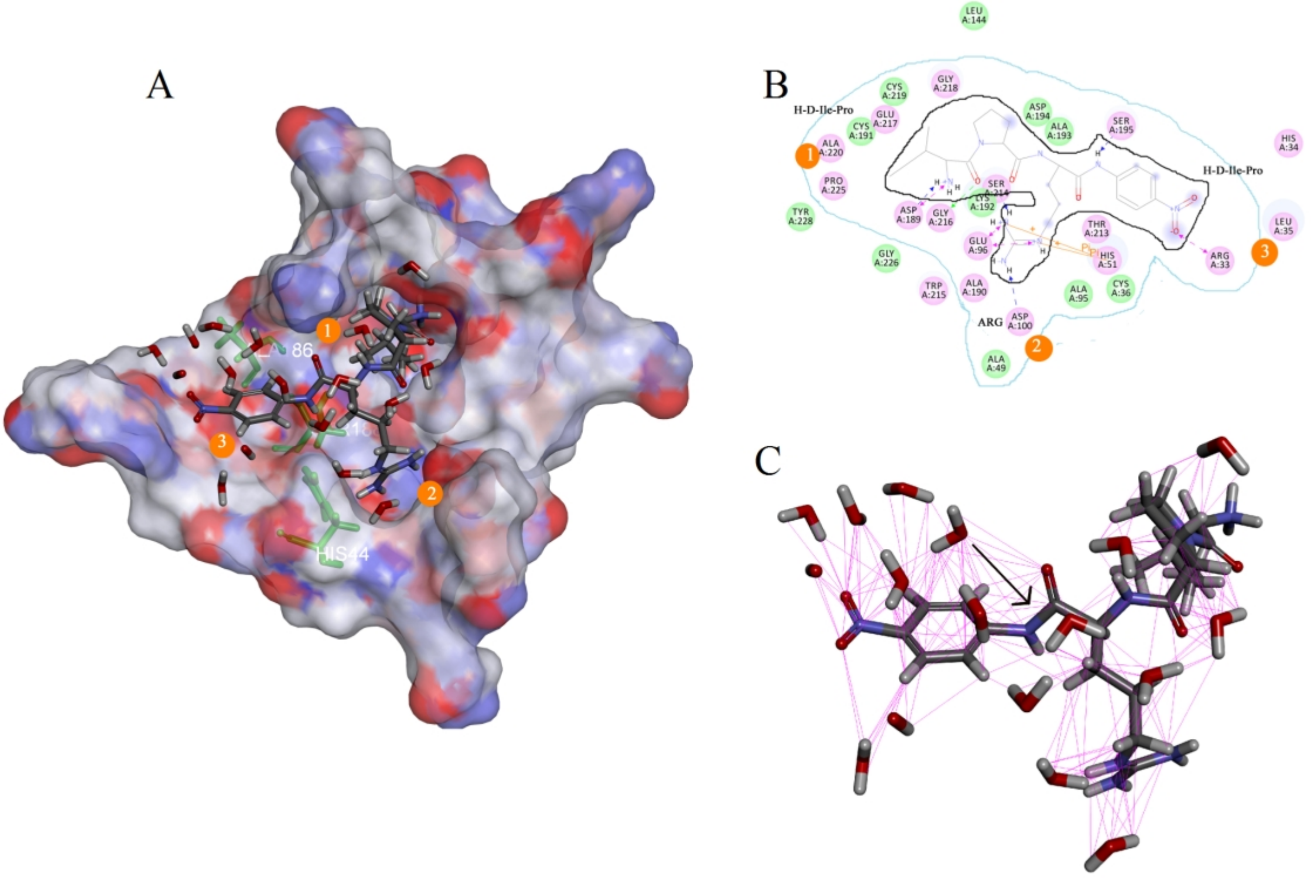

Fig. S2. Post simulation results of E-S complex. A. The surface model of the enzyme complexed with S-2288 wherein the encircled numbers represent the 3 pockets of the substrates.B. The enzyme-substrate interactions are represented in 2D format. Interacting residues are displayed as colored discs wherein residues involved in hydrogen-bond, charge or polar interactions are represented by pink circles and those with specific charge interactions are represented by a pink dashed arrow with heads on both sides. Residues involved in van der Waals interactions are represented by green circles. Hydrogen-bond interactions with amino acid side-chains are represented by a blue dashed arrow directed towards the electron. Charge interactions are represented by a pink dashed arrow with heads on both sides. The solvent accessible surface is shown as a diffuse background circle with the radius proportional to the exposure. H-D-Ile-Pro binds to pocket 1, Arg of the substrate binds to pocket 2 and p-nitrolinamide binds to Pocket 3. Water moclecules that are close to the susbtrate specifically nitroamilide are shown using arrows in C.

Supplementary Figure 3

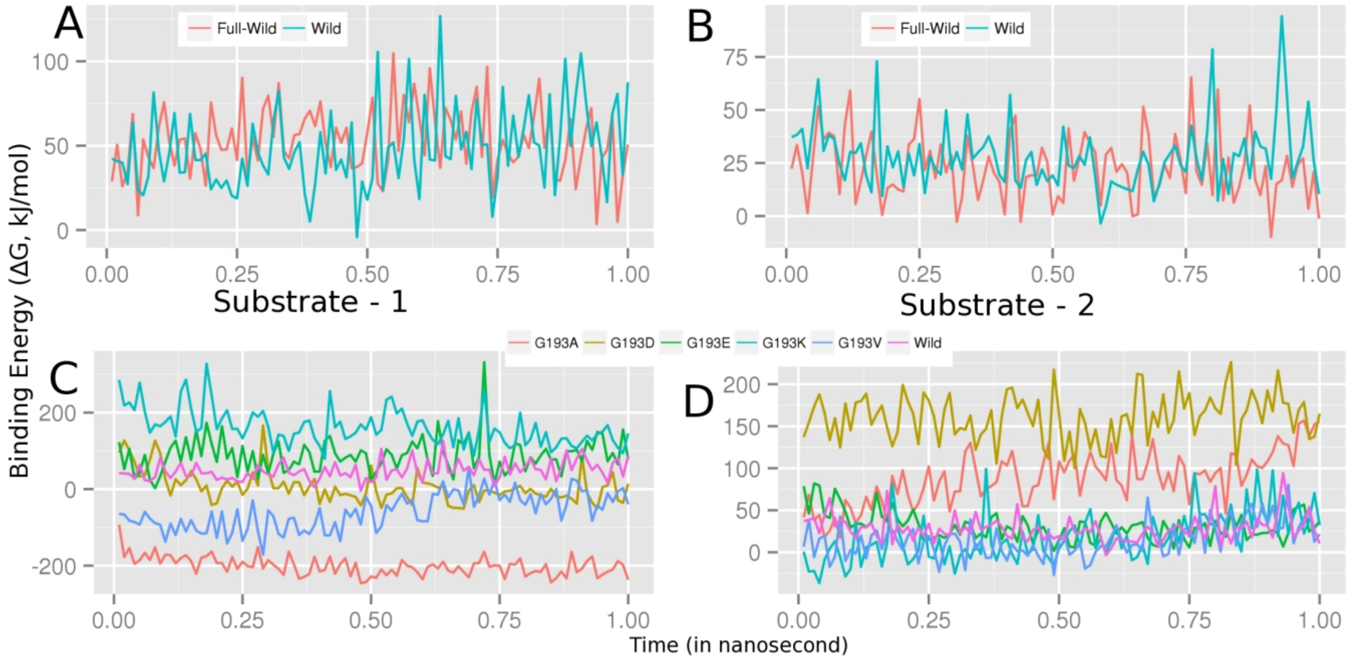

Fig. S3. Molecular mechanics Poisson–Boltzmann surface area (MM-PBSA), a method to estimate interaction free energies was calcuated between the substrates and the residues within 6Å radius of the binding site. A and B were computed for the wild type enzyme wherein the full protein is compared with the residues within 6Å radius of the susbtrate that was used for the simulations. MM-PBSA calcuations show similar binding enegies for the full and the partial protein revaling that there is no denaturation caused in the partial protein within 1 ns of simulation. The same is true with the mutant enzyme variants simualted as partial protein (C and D)

Supplementary Figure 4

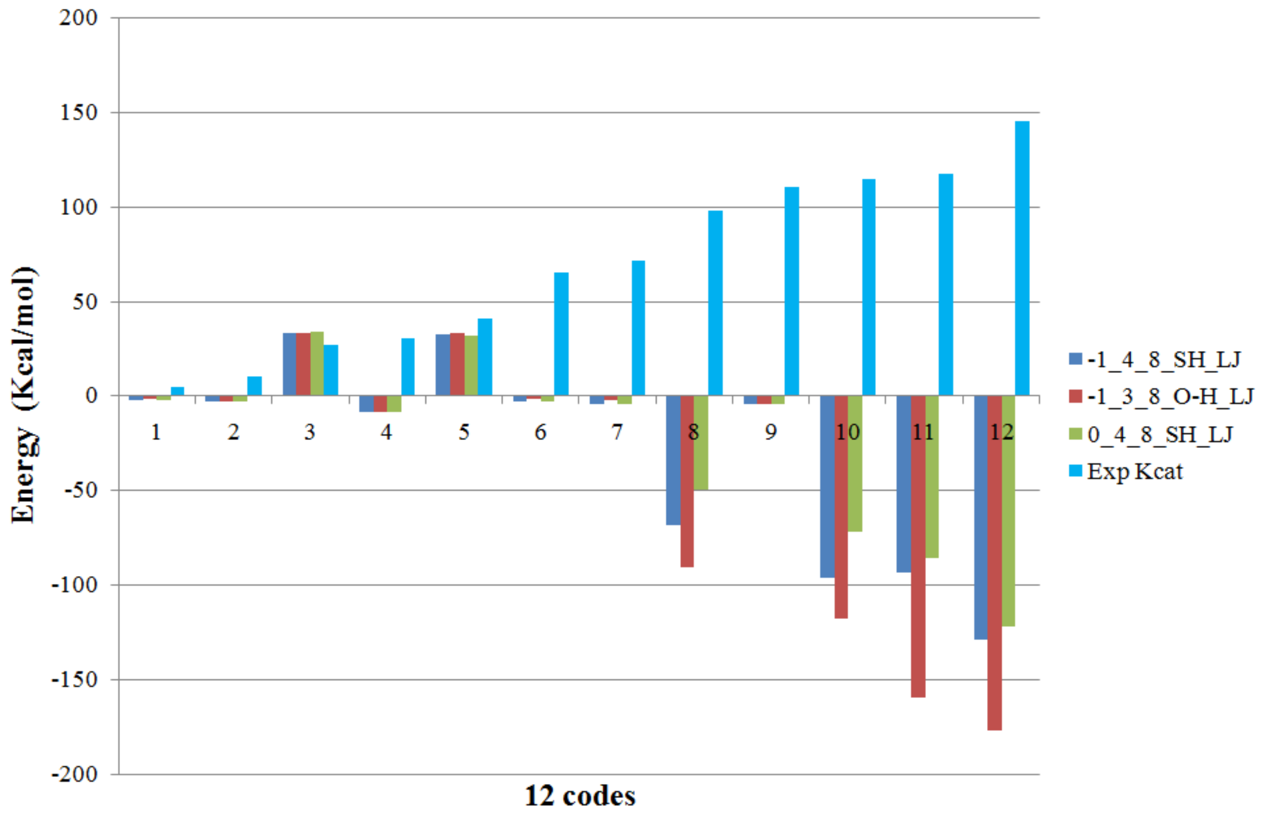

Fig. S4. Graph of the LJ energy values stored in few of the IEDs and the activity values of the 12 codes. It is observed that as the energy values decreases the activity of the codes increases, signifying a negative correlation. The keys in the graph represent the IEDs in such a way that the first three numbers separated by underscores represents the grid points (x, y, z coordinate) in the grid that stores the computed energy values. The proceeding characters are the name of the probe atom that is used to compute the energy of interaction between the CEP and itself. The last character represents the kind of interactions, Lennard-Jones interactions (LJ) or Coulombic interactions (C).

Supplementary Figure 5

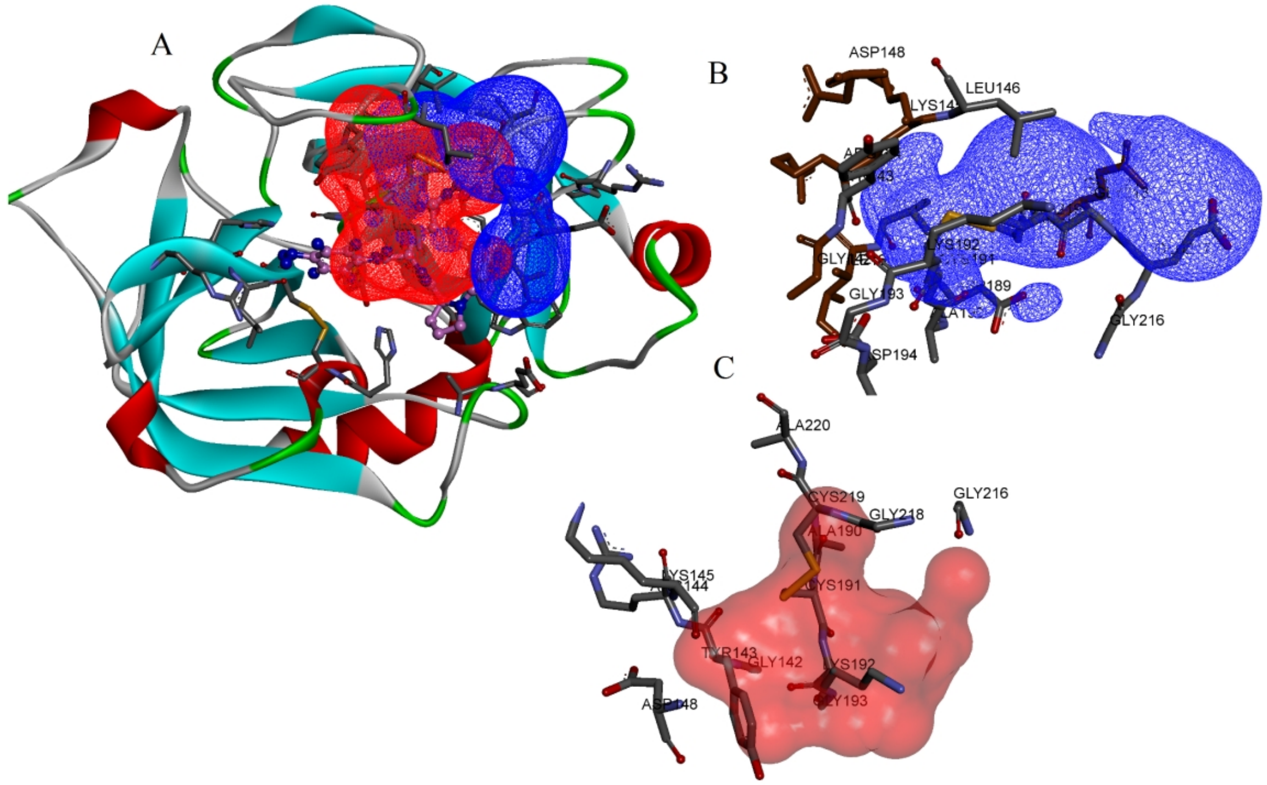

Fig. S5. IEDs mapped on the active site that correlate with the enzyme activity. A. Residues close to the IEDs with negative (Blue) and positive (Red) *r* values. B. Residues close to the IEDs with negative *r* values. C. Residues close to the IEDs with positive *r* values. The residues close to the IDEs with Negative *r* values states that the activity values of the enzymes increases as the energy values stored in different IEDs decreases. Lower energy values suggest stable intermolecular interactions.

Supplementary Figure.6

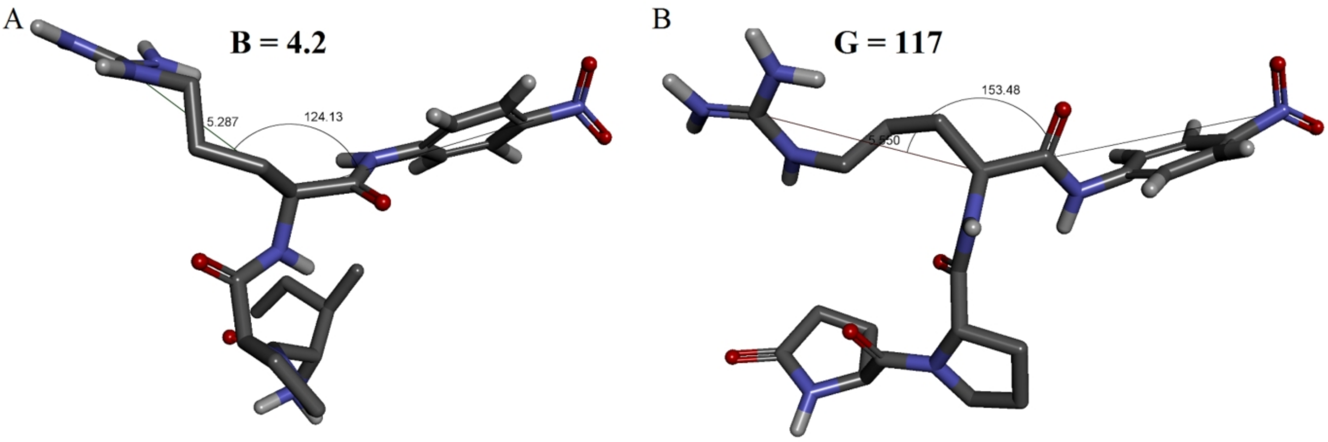

Fig. S6. The binding conformations of the substrates in the active site of the enzyme differ with mutants with high and low activity. Figure shows the binding conformation of the substrates S-2288(A) and S-2366 (B) in the enzyme mutants that contain mutations Asp (low activity) and Ala (high activity) at position 193, respectively. A “T” conformation of the substrate is observed in the mutants showing high activity and this conformation is slightly distorted to a ‘Y” conformation in the enzyme mutants with low activity. There is an angle difference of 30° between the chiral carbon and the two end atoms of Arg and nitrolinamide of the substrates. The conformation between the chiral carbon and the end atom nitrogen of Arg of the substrates is comparatively linear in mutant enzymes with higher activity. This may be due to the substitutions with bulkier side chains at position 193 that would sterically hinder the normal binding mode of Arg of the substrate.

Supplementary Tables

Table. S1. Docking results of the two substrates against different mutant enzymes and the wild type enzyme. The capitalized alphabets in brackets in the first column represent mutations at positions 193 of FXIa. The docked conformations were selected based on the atomic distance between Ser195 and His57 which is shown in the last column. The distances between the OH atom of Ser195 and carbonyl carbon of the substrate and the nitrogen (N1) of His57 and the amide nitrogen of the peptide bond between Arg and nitroanilide of the substrate were measured to select the substrate binding modes.

Table. S2. CSV file containing all the IEDs of the 12 codes. The first column represents the names of the IEDs wherein the first three numbers separated by underscores represent the grid points (x, y, z coordinate) in the grid that stores the computed energy values. The proceeding characters are the name of the probe atom that was used to compute the energy of interaction between the CEP and itself. The last character represents the kind of interactions, Lennard-Jones interactions (LJ) or Coulombic interactions (C). The first row represents the code given to a specific E-S reaction as per Table. 1. The values in the file represent the intermolecular interaction energies (kcal/mol) between the CEP and the probe atoms calculated by LQTAgrid.

Table. S3. Table shows the correlation values of each IED's against the activity value. The first column represents the names of the IEDs wherein the first three numbers separated by underscores represent the grid points (x,y,z coordinate) in the grid that stores the computed energy values. The second column represents the correlation coefficients of the respective IDE's against the activity values

Table. S4. Frequency table of the correlation studies between 156, 250 IEDs of the 12 codes and the activity values of the enzymes. It can be observed that nearly 6198 IEDs are showing *r* value greater than 0.45 against the activity values.

Table. S5. Table shows q^2, $r^2$and *RMSE* values of the 120 QSAR models derived using 1875 IDEs. The combination used in the test set are given as model codes, for example the code "ABC" is given to the model that contains codes A and B in the test set and codes E, F, H, I, J, K & L in the training set.

Table. S6. q^2, $r^2$and *RMSE* values of the 120 QSAR models derived using 6198 IDEs. The combination used in the test set are given as model codes, e.g. the code "ABC" is given to the model that contains codes A and B in the test set and codes E, F, H, I, J, K & L in the training set.

Tables. S7, S8, S9, S10, S11 & S12. Microsoft Excels containing the PLS regression models of the codes HIL, CFJ, ABL, FJK, BCF and ABH. The intercept and the regression coefficients of the PLS models given in the column "P" were used to derive the predicted activity for the test sets and external

validation set. Column B to M contains the energy values of IEDs of the respective codes given as column headings. Row “A” contains the name of the IEDs wherein the first three numbers separated by underscores represents the grid points (x, y, z coordinate) in the grid that stores the computed energy values. The proceeding characters are the name of the probe atom that was used to compute the energy of interaction between the CEP and itself. The last character represents the kind of interactions, Lennard-Jones interactions (LJ) or Coulombic interactions (C). Column R to AC stores the product of coefficients and the respective energy values of a specific code. The sum of any of these columns from R to AC including the intercept of the PLS equation gives the predicted activity values which is given in the last row.

Table. S13. q^2, $r^2$and *RMSE* values of the 120 QSAR models derived using 19764 IDEs. The combination used in the test set are given as model codes, for example the code “ABC” is given to the model that contains codes A and B in the test set and codes E, F, H, I, J, K & L in the training set.

Table. S14 Specificity and sensitivity tests of the QSAR models**.**
